# Supplementary material for: Lifestyle Habits Predict Academic Performance in High School Students: The Adolescent Student Academic Performance Longitudinal Study (ASAP)
Source: Int J Environ Res Public Health. 2019 Dec 29;17(1):243. doi: 10.3390/ijerph17010243 (PMC6982263; doi:10.3390/ijerph17010243)
Supplement: Supplementary file 1 [file ijerph-17-00243-s001.pdf]

Supplementary 1

Hierarchical regression analyses with theoretically-driven covariates included in the analyses, which represent all the variables measured in this study (physical activity, sleep habits, screen time and eating habits). Because of the limited sample size, we had to test separately each variable and, thereafter, we were able to test a combination of factors based on significant variables for each dependant variable.

Table S1. Hierarchical regression analysis regarding independent predictors of cognitive control in high school female students

Dependent variables	Independent variables	***β***	Total r^2	***P* value**
ΔFlanker congruent MRT	Social media on WD at Y1	0.28	0.147	0.001
	Daily meals at Y1	-0.29		
ΔFlanker incongruent MRT	Daily meals at Y1	-0.24	0.099	0.012
	Social media on WD at Y1	0.22		
Δ1-back accuracy	Daily meals at Y1	0.39	0.210	0.000
	ΔTotal screen on WD	-0.24		
Δ2-back accuracy	ΔVideo games on WE	-0.32	0.100	0.007
Δ2-back MRT	Social media on WD at Y1	0.41	0.206	0.000
	Daily meals at Y1	-0.25		

MRT: mean reaction time, WD: weekdays, WE: weekend, Y1: Year 1. None of the demographics variables (age, pubertal status and socioeconomic status) included in step 1 analysis remained in the final model. None of the lifestyle variables remained as a significant predictor for Δ in Flanker congruent accuracy, Δ in Flanker incongruent accuracy, Δ in 1-back MRT and Δ in d'.

Table S2. Hierarchical regression analysis regarding independent predictors of cognitive control in high school male students

Dependent variables	Independent variables	*β*	Total r^2	*P* value
Δ2-back accuracy	ΔSocial media on WE	-0.28	0.081	0.024

MRT: mean reaction time, WD: weekdays, WE: weekend, Y1: Year 1. None of the demographics variables (age, pubertal status and socioeconomic status) included in step 1 analysis remained in the final model. None of the lifestyle variables remained as a significant predictor for Δ in Flanker congruent accuracy, Δ in Flanker incongruent accuracy, Δ in Flanker congruent MRT, Δ in Flanker incongruent MRT, Δ in 1-back accuracy, Δ in 1-back MRT, Δ in 2-back MRT and Δ in d’.

Table S3. Hierarchical regression analysis regarding independent predictors of academic performance in high school female students

Dependent variables	Independent variables	**β**	Total r^2	*P* value
ΔLanguage	ΔWake-up time on WE	0.25	0.062	0.03

WE: weekend. None of the demographics variables (age, pubertal status and socioeconomic status) included in step 1 analysis remained in the final model. None of the lifestyle variables remained as a significant predictor for Δ in overall average, Δ in science, Δ in mathematics.

Table S4. Hierarchical regression analysis regarding independent predictors of academic performance in high school male students

Dependent variables	Independent variables	*β*	Total r^2	*P* value
ΔOverall average	ΔDaily servings of F/V	0.44	0.334	0.000
	ΔBedtime on WD	-0.35		
ΔScience	Age	0.60	0.392	0.000
	ΔStudying time	0.24	0.059	0.009
ΔMathematics	Age	-0.26	0.069	0.027
	ΔBedtime on WD	-0.45	0.202	0.000
ΔLanguage	ΔComputer on WE	-0.39	0.179	0.001
	Video games on WE at Y1	-0.25		

F/V: fruits and vegetables, WD: weekdays, WE: Wweekend. None of the demographics variables (age, pubertal status and socioeconomic status) included in step 1 analysis remained in the final model for Δ in overall average and Δ in in language.
